# Supplementary material for: Unveiling the Bioactive Potential of the Invasive Jellyfish Phyllorhiza punctata Through Integrative Transcriptomic and Proteomic Analyses
Source: Biomolecules. 2025 Aug 4;15(8):1121. doi: 10.3390/biom15081121 (PMC12383608; doi:10.3390/biom15081121)
Supplement: Supplementary file 1 [file biomolecules-15-01121-s001.zip › Suplementary Material/FileS3/PEPFOLD4/XP_065067169_11080/clusters.html]

 PEP-FOLD clusters


Download archive of all models  here   

|  |  |  |
| --- | --- | --- |
| # | file | sOPEP |
| # cluster 1 - Size: 1 |  |  |
|  | XP\_065067169\_11080-model1 | -26.0569 |
| # cluster 2 - Size: 1 |  |  |
|  | XP\_065067169\_11080-model2 | -25.7407 |
| # cluster 3 - Size: 9 |  |  |
|  | XP\_065067169\_11080-model3 | -16.489 |
|  | XP\_065067169\_11080-model3.1 | -15.8532 |
|  | XP\_065067169\_11080-model3.2 | -15.1823 |
|  | XP\_065067169\_11080-model3.3 | -14.7751 |
|  | XP\_065067169\_11080-model3.4 | -11.7132 |
|  | XP\_065067169\_11080-model3.5 | -11.307 |
|  | XP\_065067169\_11080-model3.6 | -10.7022 |
|  | XP\_065067169\_11080-model3.7 | -10.6605 |
|  | XP\_065067169\_11080-model3.8 | -9.79644 |
| # cluster 4 - Size: 1 |  |  |
|  | XP\_065067169\_11080-model4 | -14.9154 |
| # cluster 5 - Size: 2 |  |  |
|  | XP\_065067169\_11080-model5 | -14.6098 |
|  | XP\_065067169\_11080-model5.1 | -14.1657 |
| # cluster 6 - Size: 2 |  |  |
|  | XP\_065067169\_11080-model6 | -14.5998 |
|  | XP\_065067169\_11080-model6.1 | -11.4192 |
| # cluster 7 - Size: 2 |  |  |
|  | XP\_065067169\_11080-model7 | -14.5665 |
|  | XP\_065067169\_11080-model7.1 | -13.0462 |
| # cluster 8 - Size: 1 |  |  |
|  | XP\_065067169\_11080-model8 | -13.733 |
| # cluster 9 - Size: 1 |  |  |
|  | XP\_065067169\_11080-model9 | -13.4725 |
| # cluster 10 - Size: 1 |  |  |
|  | XP\_065067169\_11080-model10 | -13.0295 |
| # cluster 11 - Size: 1 |  |  |
|  | XP\_065067169\_11080-model11 | -12.49 |
| # cluster 12 - Size: 5 |  |  |
|  | XP\_065067169\_11080-model12 | -12.1116 |
|  | XP\_065067169\_11080-model12.1 | -9.09326 |
|  | XP\_065067169\_11080-model12.2 | -9.04372 |
|  | XP\_065067169\_11080-model12.3 | -8.80115 |
|  | XP\_065067169\_11080-model12.4 | -7.72148 |
| # cluster 13 - Size: 11 |  |  |
|  | XP\_065067169\_11080-model13 | -11.5494 |
|  | XP\_065067169\_11080-model13.1 | -10.7103 |
|  | XP\_065067169\_11080-model13.2 | -10.4715 |
|  | XP\_065067169\_11080-model13.3 | -10.3431 |
|  | XP\_065067169\_11080-model13.4 | -9.90602 |
|  | XP\_065067169\_11080-model13.5 | -9.90078 |
|  | XP\_065067169\_11080-model13.6 | -9.59318 |
|  | XP\_065067169\_11080-model13.7 | -9.52122 |
|  | XP\_065067169\_11080-model13.8 | -8.48887 |
|  | XP\_065067169\_11080-model13.9 | -8.16483 |
|  | XP\_065067169\_11080-model13.10 | -7.81995 |
| # cluster 14 - Size: 1 |  |  |
|  | XP\_065067169\_11080-model14 | -11.443 |
| # cluster 15 - Size: 2 |  |  |
|  | XP\_065067169\_11080-model15 | -11.3754 |
|  | XP\_065067169\_11080-model15.1 | -9.47245 |
| # cluster 16 - Size: 3 |  |  |
|  | XP\_065067169\_11080-model16 | -10.9596 |
|  | XP\_065067169\_11080-model16.1 | -9.80481 |
|  | XP\_065067169\_11080-model16.2 | -8.58763 |
| # cluster 17 - Size: 3 |  |  |
|  | XP\_065067169\_11080-model17 | -10.33 |
|  | XP\_065067169\_11080-model17.1 | -9.46898 |
|  | XP\_065067169\_11080-model17.2 | -7.76079 |
| # cluster 18 - Size: 3 |  |  |
|  | XP\_065067169\_11080-model18 | -10.1171 |
|  | XP\_065067169\_11080-model18.1 | -9.8037 |
|  | XP\_065067169\_11080-model18.2 | -9.02944 |
| # cluster 19 - Size: 1 |  |  |
|  | XP\_065067169\_11080-model19 | -10.0084 |
| # cluster 20 - Size: 1 |  |  |
|  | XP\_065067169\_11080-model20 | -9.78178 |
| # cluster 21 - Size: 1 |  |  |
|  | XP\_065067169\_11080-model21 | -9.7815 |
| # cluster 22 - Size: 1 |  |  |
|  | XP\_065067169\_11080-model22 | -9.75254 |
| # cluster 23 - Size: 1 |  |  |
|  | XP\_065067169\_11080-model23 | -9.62596 |
| # cluster 24 - Size: 4 |  |  |
|  | XP\_065067169\_11080-model24 | -9.51544 |
|  | XP\_065067169\_11080-model24.1 | -9.3331 |
|  | XP\_065067169\_11080-model24.2 | -8.85122 |
|  | XP\_065067169\_11080-model24.3 | -8.78271 |
| # cluster 25 - Size: 2 |  |  |
|  | XP\_065067169\_11080-model25 | -9.514 |
|  | XP\_065067169\_11080-model25.1 | -8.49173 |
| # cluster 26 - Size: 3 |  |  |
|  | XP\_065067169\_11080-model26 | -9.47977 |
|  | XP\_065067169\_11080-model26.1 | -7.98601 |
|  | XP\_065067169\_11080-model26.2 | -7.60528 |
| # cluster 27 - Size: 2 |  |  |
|  | XP\_065067169\_11080-model27 | -9.2567 |
|  | XP\_065067169\_11080-model27.1 | -7.45615 |
| # cluster 28 - Size: 2 |  |  |
|  | XP\_065067169\_11080-model28 | -9.22791 |
|  | XP\_065067169\_11080-model28.1 | -8.0801 |
| # cluster 29 - Size: 1 |  |  |
|  | XP\_065067169\_11080-model29 | -8.98695 |
| # cluster 30 - Size: 2 |  |  |
|  | XP\_065067169\_11080-model30 | -8.8923 |
|  | XP\_065067169\_11080-model30.1 | -8.54409 |
| # cluster 31 - Size: 3 |  |  |
|  | XP\_065067169\_11080-model31 | -8.59999 |
|  | XP\_065067169\_11080-model31.1 | -7.84573 |
|  | XP\_065067169\_11080-model31.2 | -7.79989 |
| # cluster 32 - Size: 5 |  |  |
|  | XP\_065067169\_11080-model32 | -8.49955 |
|  | XP\_065067169\_11080-model32.1 | -7.7958 |
|  | XP\_065067169\_11080-model32.2 | -7.52265 |
|  | XP\_065067169\_11080-model32.3 | -7.39745 |
|  | XP\_065067169\_11080-model32.4 | -7.30581 |
| # cluster 33 - Size: 2 |  |  |
|  | XP\_065067169\_11080-model33 | -8.39998 |
|  | XP\_065067169\_11080-model33.1 | -8.35995 |
| # cluster 34 - Size: 1 |  |  |
|  | XP\_065067169\_11080-model34 | -8.34168 |
| # cluster 35 - Size: 8 |  |  |
|  | XP\_065067169\_11080-model35 | -8.31416 |
|  | XP\_065067169\_11080-model35.1 | -8.10847 |
|  | XP\_065067169\_11080-model35.2 | -8.04285 |
|  | XP\_065067169\_11080-model35.3 | -7.88412 |
|  | XP\_065067169\_11080-model35.4 | -7.76426 |
|  | XP\_065067169\_11080-model35.5 | -7.73038 |
|  | XP\_065067169\_11080-model35.6 | -7.71658 |
|  | XP\_065067169\_11080-model35.7 | -7.38184 |
| # cluster 36 - Size: 2 |  |  |
|  | XP\_065067169\_11080-model36 | -8.29852 |
|  | XP\_065067169\_11080-model36.1 | -7.97892 |
| # cluster 37 - Size: 1 |  |  |
|  | XP\_065067169\_11080-model37 | -8.21373 |
| # cluster 38 - Size: 1 |  |  |
|  | XP\_065067169\_11080-model38 | -8.20974 |
| # cluster 39 - Size: 2 |  |  |
|  | XP\_065067169\_11080-model39 | -8.10896 |
|  | XP\_065067169\_11080-model39.1 | -7.93847 |
| # cluster 40 - Size: 2 |  |  |
|  | XP\_065067169\_11080-model40 | -8.0089 |
|  | XP\_065067169\_11080-model40.1 | -7.56687 |
| # cluster 41 - Size: 1 |  |  |
|  | XP\_065067169\_11080-model41 | -7.9684 |
| # cluster 42 - Size: 1 |  |  |
|  | XP\_065067169\_11080-model42 | -7.81453 |
|  |  |  |
